# Secret groups and open forums: Defining online support communities from the perspective of people affected by cancer

**DOI:** 10.1177/2055207619898993

**Published:** 2020-01-16

**Authors:** Lydia Jo Harkin, Kinta Beaver, Paola Dey, Kartina Aisha Choong

**Affiliations:** 1 Psychology Department, Nottingham Trent University, UK; 2 School of Sport and Health Sciences, University of Central Lancashire, UK; 3 Medical School, Edge Hill University, UK; 4 School of Law and Social Sciences, University of Central Lancashire, UK

**Keywords:** eHealth, cancer, online community, social media, social support

## Abstract

**Objective:**

A quarter of people diagnosed with cancer lack social support. Online cancer communities could allow people to connect and support one another. However, the current proliferation of online support communities constitutes a range of online environments with differing communication capacities and limitations. It is unclear what is perceived as online cancer community support and how different features can help or hinder supportive group processes. This study aimed to explore how perceived support is influenced by the different features and formats of online support environments.

**Methods:**

In-depth qualitative interviews were conducted with 23 individuals affected by a range of cancer diagnoses, including both cancer survivors and family members. Data were analysed using deductive thematic analysis guided by a constructivist epistemological perspective.

**Findings:**

Online supportive communities were defined and differentiated by two themes. Firstly, ‘Open forums’ were identified with thematic properties which facilitated a uniquely informative environment including ‘Safety in anonymity’, ‘Perceived reliability’ and ‘Exposure and detachment’. Secondly, ‘Secret groups’ were identified with thematic properties which enhanced an emotionally supportive environment including ‘Personalised interactions’, an overt ‘Peer hierarchy’, and ‘Crossing the virtual divide’.

**Conclusions:**

Properties of groups can engender different degrees of interpersonal relations and different supportive interactions. In particular, support community designers may want to adapt key features such as anonymity, trustworthiness of websites, and the personalised nature of conversations to influence the development of supportive environments. In personalised peer-led groups, it may be prudent to provide guidance on how to reassert a positive environment if arguments break out online.

## Introduction

In the UK, it is estimated that over half of adults will be diagnosed with cancer during their lifetime.^1^ The illness journey is commonly associated with uncertainty regarding the diagnosis, treatment and recovery or illness progression.^2^ The diagnosis is stigmatising, which can have negative implications for patients’ social relationships and psychological adjustment.^3^ Thus, the UK and the US cancer clinical care guidelines often recommend additional care resources such as peer support groups to assist self-management of cancer concerns and non-clinical levels of distress.^4,5^ Peer support groups can help to ease patient distress via regular social interactions and emotional support shared with fellow cancer survivors.^6,7^ Psychological social cure theory proposes that identifying with a group of like-minded people helps individuals adapt to the role of illness in their lives, acting as a buffer from stressful life events and becoming a ‘social cure’ to ease distress.^8^ However, face-to-face cancer support groups tend to have low attendance and high drop-out rates.^9^ Thus, there are increasing numbers of adults who require a more accessible mode of psychosocial support for cancer.

Online peer support may be a viable resource for people affected by cancer in the Western world. In 2018, 89% and 90% of adults in the USA and UK have Internet access respectively.^10,11^ Internet sites are a popular resource for health information and support.^12^ In a French study, 85% of cancer survivors regularly participated in online activities such as online health communication.^13^ Similarly, an in-clinic survey of US-based cancer survivors found approximately 68% of individuals had conducted some form of online social engagement related to their cancer, including seeking out social connections online and participating in online supportive cancer communities.^14^ Online communication does not require individuals to travel or attend a meeting at a particular time. Thus, online communities can be convenient for people homebound after cancer treatment or located in remote rural settings.^15^ Moreover, the online disinhibition effect posits that, as facial and social cues are absent on the Internet, people feel an increased freedom during written expression in this media.^16,17^ Thus, online cancer support groups may foster a unique openness in communicating about illness experience, which in turn may engender an informative, understanding and supportive response from peers.^18,19^

The evidence base regarding the effectiveness of online cancer support is modest but positive.^20^ Online community use has been reported as a positive experience amongst small samples of cancer survivors in controlled community settings.^21,22^ Online community participation has been linked to increased positive coping.^23,24^ Communities allow individuals to share information resources and discuss their treatment-related decisions.^25^ Therefore, communities may act as an additional health engagement resource in oncology.^26^ Furthermore, content analyses of existing groups reveal that individuals discuss a range of emotive experiences including treatments, risks, side effects and personal impacts of cancer such as family or psycho-sexual concerns.^25,27^ Thus, online cancer communities appear to allow individuals to actively engage with their cancer care, and to discuss potentially stigmatising concerns with a group of similarly-situated individuals.^28^

There is a vast range of online cancer communities. Much of the controlled research into such communities utilises small online groups, in environments designed to encourage participation, and with a dedicated clinical specialist moderator.^24,29^ However, online peer communities for cancer are proliferating on public webspaces which often do not utilise a clinically trained moderator. They aim to attract a wide audience of different patient and family groups, and vary in features and environments. A 2011 search of Facebook’s breast cancer-related groups found 620 peer-led support groups containing over one million members.^30^ More recently, emerging social media sites such as Twitter, have been associated with international conversations (via hashtags) to garner interest and support for cancer relevant topics from a potential pool of 330 m monthly active users.^31^ These sites present patients and healthcare professionals with the challenge of wading through an increasing number of digital resources in order to find support. Indeed, there is still no commonly used definition for an online cancer support community. A theoretical review of online peer support from a health informatics perspective suggested that the quality of cancer support online can vary dramatically according to key community features.^32^ Discussion content is shaped by features such as member characteristics, gender, age and disease group.^32^ Moreover, this may result in groups sharing different messages of support and information.^33–35^ However, there are few insights into how the online community features may be experienced from the perspectives of people affected by cancer, as analyses have often been aimed at the level of the posts which can be viewed online,^33,34^ rather than perspectives of the individual living with and adapting to cancer. It would be naïve to assume that online communities that support different types of communication and membership can provide the same forms or experiences of support. To the best of our knowledge, there have hitherto been no empirical explorations of how cancer survivors perceive the variety of available online cancer communities, and how community features may shape perceived support.

The present study aims to firstly understand what people affected by cancer perceive as online cancer community support. Secondly, it seeks to explore how perceived support is influenced by the different features and formats of online support environments.

## Methods

### Study design

This was a qualitative semi-structured interview study with people affected by cancer who used online cancer communities. To elicit a rich view of digital communities and the support they offer, we sought participants with a range of digital community experiences, recruiting by advertising the study across 19 online cancer communities on a range of platforms, including social media pages and dedicated cancer community pages. Telephone and face-to-face interview data were originally collected in 2016 for a theoretical analysis of online community use in the context of the cancer patient trajectory, and the methods of data collection have been published elsewhere.^28^ As such, the initial interview schedule, developed in consultation with the literature, was designed to elicit experiences of online community support. After conducting initial interviews, it became apparent that participants perceived the form and format of online communities pertinent to their online support experiences. Thus, the interview schedule evolved from questions such as ‘Can you tell me about using an online group for cancer’ to ‘Can you tell me about the online groups for cancer you have used?’. Furthermore, this analysis was designed to enlighten us on the previously unexplored perceived differences in experiences according to online community features. Braun and Clarke’s inductive thematic analysis approach was selected for the present research to provide an inductive approach to understanding community user perceptions, while remaining at the interpretive level of highlighting the common features from the communities which were salient to participant experience.^36^

### Participants

Twenty-three individuals were interviewed, with interviews lasting an average of 69 min (range = 43–123 min, median = 64 min). The sample ranged in age from 31–70+ years (median age = 50, mean age = 50). The majority of participants accessed online cancer communities because they were living with a personal cancer diagnosis. The sample also included two participants who used online communities as a family member affected by cancer, and three individuals who were affected by both their own and a family member’s cancer. The most common diagnoses were melanoma, breast and ovarian cancers. Table 1 summarises the characteristics of the participant sample.

**Table 1.** Participant demographic information.^1^

Participant demographics	*n = *23
Age (years)	
Mean	50
Median	50
Range	31–70+
Relationship to cancer	
Cancer survivor	18
Family member	2
Both cancer survivor and family member	3
Cancer location/type	
Skin	7
Ovary	6
Breast	5
Bowel	2
Prostate	2
Brain	1
Head and neck	1
Lung	1
Non-Hodgkin’s lymphoma	1
Pancreas	1
Sarcoma	1
Thyroid	1
Age (years)	
<31	0
31–40	4
41–50	8
51–60	5
61–70	5
0>	1
Gender	
Female	19
Male	4
Ethnicity	
White British	22
Other	1

1 Reproduced with author permissions^28^.

### Data analysis

QSR NVIVO software was used to store and manage the verbatim transcribed interview data. The data was analysed using the six phases of thematic analysis, as recommended by Braun and Clarke.^36^ This allowed the experiential information to be explored at the essentialist level, i.e. when considering the existing structures and formats of the virtual communities.^37^

Analysis was conducted in six stages.^36^ Firstly, LH, the primary analyst and the interviewer, became familiarised with the data by conducting the interviews, transcribing them and noting down initial ideas. Secondly, initial codes were developed with each individual transcript, using a line by line approach on each transcript between each interview. Thirdly, the initial codes were considered across transcripts in a search for themes. Fourthly, the themes were reviewed by the research team as a whole, before the fifth phase of definitively naming the themes. Writing and refining this article determined the final stage of thematic analysis.

### Ethics

Written informed consent was obtained from all individual participants included in the study in the form of information sheets accompanied by signed consent forms. To assure participant anonymity and online community protection, all identifying features were removed from the transcripts, including participant names, usernames and names of the digital communities. Additionally, participants were referred to according to identification codes to ensure participant anonymity. The identification code is presented below each quotation and indicates the participant number, gender (male (M) or female (F)) and their relationship to cancer (as either someone living with a diagnosis or a family member). Thus participant one’s code is Participant 1/F/Diagnosed. Ethical approval for this study was granted prior to data collection from the institutional ethics committee (approval reference: STEMH 248).

## Results

### Defining an online support community

The following findings were drawn from participants who had diverse experiences with cancer, affected by a range of diagnoses and included both survivors and family members. While the motivations for using the online communities differed according to the individual’s trajectory with cancer (analysis of which has been published elsewhere),^28^ the participants expressed common perceptions of the features of online communities. It was further noted that the participants were all familiar with the Internet and had used various cancer-related Web and social media sites during their cancer journey. Through personal trial and error, participants had established a definition of what a website or social media site must contain to constitute an ‘online support community’. Communities were websites with a purpose of hosting regular, sustained interactions. Interactions often included signposting other community members to further cancer information, venting frustrations and offering empathy amongst the virtual group. This richness of community experience was seemingly only possible on websites which focused on shared discussions. The participants rejected the idea of ‘communities’ forming on websites which focused on individuals’ journeys, such as personal blogs or websites belonging to people affected by cancer. Similarly, microblogging (more commonly known as Twitter) gave most participants the impression of messages posted by users to share their individual thoughts, rather than hosting discussions or posing questions and receiving informative answers or resources. The participants in this study found that such an individualistic focus was not conducive to developing a sustained dialogue between people affected by cancer. Therefore, participants gained no sense of ‘community’ from blogs and Twitter.

I think the blog is just me putting stuff out there. I do get people tweeting or commenting on the blog … but it’s all different people … it’s less of a community. But I think on [online community name], you know, it’s a group, there’s only us in it, you do feel like a little group, yes. (Participant 1/F/Diagnosed)

A sense of community could be achieved in a range of online groups which were framed for different memberships. For instance, communities could be designed for individuals with similar cancer diagnoses and cancer survivors with similar personal circumstances, such as parents with cancer or survivors under the age of 50 years. Though participants were sometimes members of different online communities, a notable divergence in experiences online distinguished two participant-derived classifications of communities: ‘open forums’ and ‘secret groups’. This division was drawn because these two types of communities provided distinct benefits and risks to people affected by cancer. Therefore, online communities have been redefined in this study according to this distinction, forming two themes with subthemes which outline the characteristics of most importance to the online cancer experience. All themes and subthemes are summarised in Table 2 and described in detail below.

**Table 2.** Thematic definition of online cancer communities.

Open forums	Secret groups
Safety in anonymity	Personalised interactions
Perceived reliability	Peer hierarchy
Exposure and detachment	Crossing a virtual divide

### Open forums

Open forums are those communities with contents in the public domain which could be found by accessing a website or performing a Google search. Open communities were often referred to as forums, and were usually hosted by well-known cancer charities. As forums were easily discovered, their audience was usually large and diverse, in order to support as many people as possible. This gave the impression of open forums as being rich in a range of messages and experiences. For those trying to find a peer affected by cancer, it could be reassuring to discover a forum online and observe many individuals sharing the cancer experience. Open forums were often discovered by international cancer survivors and messages were posted from a range of time zones, which allowed individual posts and responses to appear in communities at all hours. The exceptions to this were the less populated forums, such as those aimed at rarer cancer diagnoses. Fellow community visitors discussed a range of different treatment pathways or offered advice which differed according to variations in international healthcare systems. Thus, larger open forums were perceived as a rich source of information, requiring greater sifting and filtering for relevance. However, juxtaposed against the information-rich environment was a greater sense of mistrust towards the intentions and empathy offered by community members in the open groups, particularly, when participants compared open groups with their private counterpart communities. The features that engendered the open communities as a particularly informative but less supportive environment are presented in the subthemes ‘Safety in anonymity’, ‘Perceived reliability’ and ‘Exposure and detachment’.

I actually, erm, was only looking at trusted information sites. I didn’t know where to go, so I was like going on to like [UK national cancer charity name, UK national ovarian cancer charity name], and that’s where I first started, knowing that they were a bit more regulated, that I wouldn’t get hopefully too scared. (Participant 13/F/Diagnosed)

### Safety in anonymity

Most open communities allowed participants to visit the groups without demonstrating their presence on the webpage or logging into an account. If individuals chose to post a message to the group, many open communities required individuals to create an account with a non-identifying username. This made open communities feel relatively anonymous both when visiting and posting messages. This feature allowed individuals to ‘lurk’ or sit on the sidelines of conversations. Participants evaluated whether the discussions could support their psychosocial needs and remained hidden and anonymous to learn the common types of interactions and terminologies used in groups. This was particularly valuable to patients or family members affected by a recent diagnosis as it allowed them to learn the language of both the community and the illness; participants could watch and decide which discussions were relevant or irrelevant to their concerns. Several participants remained anonymous, even after years of visiting the community pages, whilst others waited a short time before feeling able to post a message anonymously. Ultimately, anonymous interactions with communities were positioned as requiring very little commitment to the other group members and activities of a community. Thus, anonymity imparted an element of emotional safety as individuals could retain an emotional distance from distressing discussions, obtain the information they needed, and leave these groups at any time.

I wanted to be convinced that it was a good place for her to be. And so I spent a while, first of all, just, I did not join but I just watched and listened, you know, to see how things went with others, to see if it was going to be a positive and up-building experience. (Participant 14/F/Family member)

If individuals did post to a community, most participants perceived these initial posts as brave, as they often conveyed a state of desperation or despair. To expose oneself in front of an open community, rather than remaining in the ‘safe’ lurking position, indicated a desperate need for support and information. Given the preference for lurking in open forums, several participants noted that it was a small number of individuals providing responses and answering questions. Indeed, two of the participants in this study were active contributors to open forums, and described the small network of regular posters online as a supportive network whom they could rely on for advice. Conversely, several participants felt that they were ignored when their messages went unanswered in open forums. This experience was particularly pertinent for the participants with a rarer form of their illness, as they believed that their experiences were different from other community members, and questioned whether they could relate to their peers in the online groups. For example, one such participant posted in a forum to request help and advice, but received no response from the community. This experience reinforced her feelings of social isolation. The format of open forums compounded this perception because she could view the many responses received by other posts.

It’s such a big thing in your life and when you put it out there and no one acknowledges. And you can see all these responses to other people’s questions, erm, that you’ve been looking at yourself. And there is something, you wonder, what is it about this and about me that people don’t want to help or they’re not interested in … it almost feels like a bit of a voyeuristic. (Participant 15/F/Both family and diagnosed)

### Perceived reliability

Cancer care organisations advertised their specialist phone-lines on forum webpages, and several forums invited trained clinicians to participate in online discussions at scheduled dates. Thus, by discovering an open community, participants were sometimes able to connect and communicate with reliable sources of cancer information. However, these phone-lines were only available for limited daytime hours, whereas participants accessed online communities at all hours of the day. Therefore, participants often used the forums as a stand-in or in preparation for discussions with healthcare professionals via the specialist phone lines or in conversation with their own specialist teams. Open communities described in this study are not moderated regularly by cancer specialists and so the validity of the majority of community information was not regularly assessed. Despite this, the open forum’s proximity to valid sources of cancer information made the discussions appear more valid and reliable. Indeed, most participants commonly described open forums as useful for obtaining what they viewed as ‘expert’ information, even if the information was obtained mainly from fellow cancer survivors. However, for participants who lived with a rarer form of their diagnoses and who struggled to obtain any information about their illness either online or through their healthcare professional team, this general trend has the opposite effect. It compounded their frustration that their diagnosis was isolating and complex.

I think the better ones are the ones like on the [well known UK cancer charity and cancer research organisations] with the most sort of health professionals, rather than just people talking … they are sort of moderated. (Participant 7/F/Diagnosed)

### Exposure and detachment

As open forums were publicly accessible, most participants were aware of the potential for intruders or malicious individuals to read the messages posted online. Although most community members were perceived as well-intentioned, most participants were wary about how much personal information was shared in the online forums. Indeed, several participants received private ‘phishing’ or hoax messages from other forum visitors. These messages drew on seemingly shared cancer experiences and aimed to persuade participants to share personal financial and identifying information. No participants were persuaded by such scams, but the presence of scams made participants re-evaluate how safe the forums would be for what were perceived as vulnerable people potentially using online communities. Thus, occurrences of scams negatively impacted the perceived benefits of online support.

You get people there, not for reasons of either giving or receiving support, some people trawling for contacts. There is a kind of Munchausen syndrome by proxy thing that goes on sometimes … in the support groups. People pretend to be, people come on sites and pretend to have cancer. (Participant 2/M/Diagnosed)

I knew then it was a scam … . It didn’t upset me. It makes me sorry somebody would come on cancer sites and do that because there are some very vulnerable people on there, which for that it makes me sad. (Participant 12/M/Diagnosed)

Many online forums warned individuals not to share identifiable information, such as names and contact details. Thus, participants commonly did not know personal information about their fellow group members and were less willing to share the fullness of their thoughts and feelings about experiences in forums. Posting to forums was described by most participants as seeking answers to specific questions, rather than sharing emotive experiences. This established an air of detachment from fellow individuals communicating in open forums. As a consequence of seeing hoax accounts online, and due to detachment from other group members, several participants stopped visiting open online support communities once they had found the information they sought or moved on to more ‘private’ forms of cancer community when seeking emotional support, as will be detailed in the next theme.

Interviewer: Were you ever aware of who certain people communicating in the forums were?

Participant: No and I wasn’t interested in that at all.

Interviewer: Why was that?

Participant: Well because I wasn’t trying to make friends [laugh]. I just wanted to know information. (Participant 10/F/Family member)

### Secret groups

Secret, or private, forms of online cancer support communities were those which prevented non-group members from viewing discussions. As a popular social media site known to almost all of the participants, Facebook was considered a convenient site to access and host online support communities. Less commonly, other sites such as Google Groups and password-protected areas of charitable cancer organisation websites also hosted secret communities. Secret groups were often created and maintained by people affected by cancer, rather than an organisation focused on cancer support. They were often devoted to one aspect of individuals’ identities, for example, there were groups for specific diagnoses, for parents living with cancer, or for cancer survivors under 30 years of age. This invitation-based membership meant that private communities ranged in size, from hundreds of members to particularly exclusive groups containing under 10 members. Furthermore, such private style of groups was described by some participants as ‘secret’ as they could only be discovered and visited by invitation. Several participants described the act of being invited into a secret group as akin to joining an exclusive group. These environments helped to engender a greater sense of shared network amongst group members when compared with open forums. Features such as ‘personalised interactions’, an overt ‘peer hierarchy’ and ‘crossing the virtual divide’ helped to identify secret groups as a setting in which emotional support could be sought and shared.

I kept hearing them talking about it on the [breast cancer forum]. They kept on about this secret network, this secret network that was on Facebook for younger people … whereas with the other sites [three open forum names] are more to me about asking a question and then providing support by being able to try to answer other people’s questions, rather than a sense of a network. (Participant 15/F//Both family and diagnosed)

### Personalised interactions

Unlike open forums, secret communities often used real names and pictures, revealing information about community members to one another such as gender, age or ethnicity. This was described as ‘putting a face to a name’ and helped to impart explicit information about fellow group members, such as age, gender and ethnicity. Facebook ‘likes’ were also a small but significant feature for engendering a greater feeling of personal support in online communities. Several participants received only a few written replies to messages, but many ‘likes’ from the community members. This simple symbol showed participants that other members were reading, appreciating and supporting their experiences. As such, most participants felt that communication in such secretive spaces became more meaningful, jovial and centred on holistic aspects of people’s families and lives, and conversations could evolve beyond discussions of the illness. This jovial atmosphere was likened by two participants to a ‘virtual bar’. Thus, participants cared more about fellow members of communities when they understood, or believed they understood, who they were. This in turn made individuals more likely to respond empathetically to those members posting emotional updates in the groups.

You felt like you’d stepped through the door of, you know, someone’s house and everyone was sort of saying hi to you … and it’s got all the additional stuff that, you know, Facebook can do. So if someone’s having a bad day, erm, or someone’s achieved something, people click like. And it’s so stupid but, you know, when you see someone has got a hundred and twenty five likes because they’ve finished chemo or, erm, I think that’s a big thing. (Participant 15/F/Both family and diagnosed)

However, almost all participants who accessed secret groups described having watched arguments unfold between group members. Several participants felt that the arguments were exacerbated by the lack of facial and tonal cues in online discussions. They felt that the type of text, capitalised words and some types of grammar could sometimes convey aggression within an online post. The private and familial nature of secret groups explicitly invited members to contribute to discussions including expressions of personal opinions. Members left candid messages to the groups about their feelings towards cancer, healthcare and charities, particularly if they felt their healthcare journey had been unfairly impacted on by an error or misjudgement. The diverse range of experiences combined with the emotive topic of the cancer journey could quickly spark arguments between members with differing opinions. Arguments unfolded slowly with individuals responding throughout a day, creating a large visual display of the arguments within the online group. This was described as upsetting for the atmosphere of the group over time. For two participants, arguments reduced the feeling of the group as a supportive space and they reduced their online activity or left the groups as a result.

There was this year a situation on one of the forum[s] that I belong [to]. And it was, and I found it quite distressing because, erm, I knew some of the people who were involved personally. And I could see how there were different points of view … . I was watching what was happening, it was like watching a sort of car crash in slow motion. And it was very difficult to know what, if anything, I could do to help resolve the situation … in the same way that if you watch people who you know have a misunderstanding that then goes, gets out and out and out of hand [sic], then it can be quite distressing. (Participant 17/F/Diagnosed)

### Peer hierarchy

Hierarchies developed within peer-led secret groups. Secret online cancer communities usually contained people affected by cancer and their families and were moderated by fellow members. Group moderators or administrators were often self-selected by individuals who set up the social media communities or were the most regular contributors to the online communities. Due to the invitation-only nature of the online communities, all moderators had the responsibility of assessing and granting new group members access to the communities. Four moderators were interviewed in this study and they variably described a range of other responsibilities including posting a welcome message to introduce new community members to groups, deleting group messages which did not adhere to community rules and removing group members who appeared to contravene the standards of the group. Participants described their responsibilities as a source of pride and self-esteem; it was gratifying to ‘give back’ to the community and be part of the support cycle. For example, 'I am an administrator for the [named] group, and the administrators filter out a lot of people who are trying to join the group for the wrong reasons' (Participant 2/M/Diagnosed).

Moderators had a powerful influence; in group disputes, the moderators’ decision was final. Their ultimate show of power was the ability to remove people from the communities. Participants who were not moderators revealed that this removal could be perceived as a striking and ‘brutal’ move, ostracising a group member from their support network. Individuals who had different opinions from the community moderators were closely watched for ‘bad behaviour’ and the power differential between group members and moderators may have been cause for concern. Thus, whilst some participants gained status in the groups, events such as enforcing the rules reminded other participants that they had a lesser level of influence over the rules and atmosphere of the online communities. Meanwhile, participants who had watched individuals being removed from groups expressed concern for their removed group members and were worried that they might be missing additional sources of support without the virtual community.

Two people were effectively, you know, let go from the site shall we say. It was all, it all got a bit unpleasant. Erm, and it was because [pause] the administrators felt that [removed group member] was promoting alternative therapies and apparently … that's one of the rules of the website, of the forum. … I wasn't aware that those people who were let go were making those claims shall we say, I didn't ever feel that anything they said was as strong as that. I wish they hadn’t been removed, because it seems a bit extreme. (Participant 3/F/Diagnosed)

### Crossing a virtual divide

The regular, personalised conversations in secret communities made it possible for members to develop friendships. Secret communities had no restrictions on sharing personal information and so participants often added members of their community as ‘Facebook friends’ and shared contact details. One socially isolated participant found that their online network was willing to attend a birthday party, and several groups hosted regular face-to-face meetings. Thus, many users of secret groups spoke of one or two members as significant friends whose interactions were important to them beyond the act of discussing cancer-related information. Several participants had removed themselves from communities over time but remained in contact with individual community members. Thus, connections developed in secret groups appeared to be more lasting than those made in open forums.

And a few of them, instead of just being on the forum, where it’s the only way I contact them, a few of them I’ve actually got them now as friends on Facebook. … But, erm, I’ve got a few of them, who are a little more than just see them online. (Participant 6/F/Diagnosed)

Not all participants benefitted from their virtual network. Emotional challenges were described when a member’s health declined or when members died from cancer. In addition, Facebook groups encroached onto participants’ ‘real’ friends and family. Several participants had attempted to hide their diagnosis and their psychosocial needs from friends and family. Whilst the content of secret online communities could remain hidden, some individuals found that their membership of a group would be shown on their Facebook profile, particularly as Facebook privacy settings altered with Facebook updates. This effectively ‘outed’ participants as a person affected by cancer to their offline social circle. Similarly, several participants described cancelling face-to-face meetings with members of their online peer network because they were embarrassed to associate with fellow cancer survivors.

I was worried that, you know, through posting on that site or even joining the group, that I would kind of be outed on my Facebook feeds to all my friends and family, which, you know, I did not even tell my family that I had cancer until three months after I started chemo, just because, you know, I was worried about how they would react. (Participant 21/F/Diagnosed)

## Discussion

This study clarifies what constitutes a contemporary online cancer community and highlights key features of communities that can influence perceived support. Previous references to online peer and health resources have considered personal websites, blogs, video diaries, Twitter, Facebook and forums as part of a homogenous set of groups.^13,19,38^ We found that a supportive sense of community was experienced in groups that specifically facilitated interactions with multiple community members. Twitter did not facilitate this sense of community, consistent with emergent research revealing that higher levels of emotional and informational support were shared in lung cancer community messages in a Facebook private group and a national charity open forum when compared with Twitter messages.^39^ Furthermore, our participants’ definition of an online community supported Rogers and Chen’s definition of online communities in 2005: as Internet groups with a shared interest, shared rules, on-going and persistent interactions, and a sense of togetherness.^40^ Many of our themes, while derived from the interviews, are consistent with extant theoretical foundations in online community interaction research. Themes of safety in anonymity, exposure and detachment online have been debated in research exploring the ‘disinhibition effect’ whereby anonymous online communities have been thought to develop differing types of interactions when compared with personalised ‘realistic’ interactions.^16,17^ Additionally, our themes concerning personalised interactions and peer hierarchy are consistent with the ‘social cure’ understanding of group interactions.^41^ That is, groups which develop group roles and hierarchies, and facilitate a shared sense of identity are likely to engender social support. Our findings offer insight into where cancer survivors and families find support online. The following paragraphs discuss the findings in relation to insights in features of, firstly, open forums and, secondly, secret groups for cancer support.

Anonymity may reduce the likelihood of emotional disclosures and offers a lesser degree of emotional support. The removal of identifying information online has been linked to the removal of social cues, such as gender and social status.^16^ According to the online disinhibition effect, fewer social cues allow for a freer communication style and richer disclosures online.^17^ In contrast, in the present study, participants found anonymity beneficial as a way to remain silent or obscured from the online community members. The greater personal information shared in secret social media-based groups created a greater emphasis on sharing and supporting group members. Social media researchers have referred to identity-centric environments as ‘nonymous’ online platforms as they show participating group members identifying features through use of real names, profiles and images.^42^ The present research demonstrates the theoretical distinction of anonymous versus nonymous online communities in a cancer community context. In such nonymous, identity-revealing platforms, identifying features, such as age, gender and culture, allow group members to have more context by which they can develop a sense of shared group identity. This gives a greater opportunity to discuss group identities, rather than personal narratives.^41^ Similarly, the social cure hypothesis posits that groups with a stronger identity and cohesion offer a greater holistic curative effect for group members.^8^ Thus, it is unsurprising that the identity-related online communities were more closely associated with a closer personal network and more readily available support, when compared with the anonymous open forums. Supportive community managers aiming to develop an emotionally supportive online community should consider the benefits of allowing group members to implicitly identify other visitors to the groups through member features, such as pictures and biographies indicating group identities.

Publicly available online communities for cancer survivors were perceived as reputable due to their association with cancer support charities and healthcare specialists. The brand of the cancer organisation or charity appears to create a supportive online ecosystem in which positive perceptions of the brand engenders a positive perception of the patient-shared information available online, and therefore enhances the likelihood of constructive community responses.^31,35^ This is likely to be a welcome finding for cancer support organisations, which aim to utilise their information portals in order to signpost families and patients to information and support services.^43,44^ Traditionally, cancer survivors and their families have reported unmet needs for information about cancer.^45,46^ As individuals become increasingly digitally active, online cancer communities could be a convenient way of signposting cancer survivors to a publicly available source of cancer information. However, our participants suggested that open communities are at particular risk from scams targeted at vulnerable cancer survivors. Reviews of available cancer community websites have shown that many are not regularly monitored for accuracy of information.^47,48^ Thus, a key recommendation for open communities is to, firstly, ensure their data security is robust and, secondly, examine ways to increase trust or allay potential fears about how patients’ online disclosures may be used.

Online communities on sites such as Facebook, which support sustainable and meaningful relationships, may be particularly valuable to socially and physically isolated individuals living with and beyond cancer.^49^ One in five cancer survivors report loneliness and a lack of social support in survivorship.^49^ However, the reliance on Facebook as a platform may be troubling. Ethico-legal challenges associated with its use include a reported privacy breach in 2018 whereupon medical support groups were automatically altered from ‘closed’ to ‘open’.^50^ These examples are a timely reminder of the challenge of hosting personal and sensitive interactions on a platform which was not designed with patient confidentiality and best practice in mind. Changes in privacy settings are likely to threaten group members’ perceived privacy and ability to disclose online,^51^ potentially undermining the online group as a source of support. This is particularly concerning considering that cancer populations are typically older adults, a population who report higher mistrust and fewer digital skills in deciphering safety and accuracy of information.^52^ Perhaps a practical way forward to avoid potential inadvertent distress from online community users on sites such as Facebook would be to develop digital literacy skills in patient populations, enabling them to evaluate the risks and benefits of differing online cancer communities.

This study revealed that the hierarchy of an online support community, particularly the intimate secret groups, may be influential with regard to the support received by the leaders, and the members. The helper-therapy principles explain that individuals helping others often experience an enhancement in self-esteem when they observe the impact of their interactions.^28^ Furthermore, as highlighted by Haslam et al. in 2018, a sense of shared identity will naturally facilitate leadership processes as individuals feel empowered to direct other group members.^8^ Positive effects of leadership occur when leaders best exemplify the group.^41^ This suggests that leadership that does not reflect the identity of the group and other group members could unravel the curative experiences of interacting with a community. Observations from the present research demonstrated that as members observed ‘poor’ behaviour from those in positions of leadership, they may have begun to associate the groups with negative behaviours. This may reflect a process known as the ‘social curse’ phenomena, in which groups may be perceived as burdensome and potentially stress-inducing, rather than a supportive resource.^53^ However, it is important to note that this influence of hierarchy, leadership and loss in online communities has been drawn from observational studies only, and future research is needed to establish whether there are health outcomes as a result of differences in perceived support amongst group moderators when compared to standard online community members. Furthermore, it is important to understand the issue of systematic group withdrawal, as this could be perceived as exclusion of members with particular perspectives and inequality of support provision.^38^ The issues of health support group arguments and appropriate leadership styles have been addressed within offline peer support contexts. National cancer charities offer information packs and training which help peer-led and professional-led support groups to establish norms and positive interactions.^54^ Considering the potential for arguments in online communities, it would be prudent to develop similar resources to engage and facilitate continued positive interactions in support groups online.

Our findings call for a critical reflection of methods which have used transcripts or analysed messages from open online communities as data.^55^ Support perceived by people affected by cancer is complex, defined by differing needs across the illness trajectory,^56^ and thus the perspectives of the individuals using the online communities are as important as examining interactions within the groups themselves. Studies have argued that it is beneficial to collect data from publicly available online communities because these groups reflect honest and natural conversations, unaffected by the presence of researchers.^57^ Whilst participant perspectives were not corroborated with real-time extracts of open versus secret communities, participants emphasised how they elected to withhold their thoughts, or to engage in personalised conversations depending on the group they used. Therefore, future Internet researchers should be aware of the nuances in online behaviours and reflect on how the context of the online world may affect or circumscribe the content posted online. Furthermore, it may be difficult to generalise psycho-social benefits which can be gained from online communities, as differently designed groups appear to be used for different purposes by cancer survivors.

As a qualitative, exploratory approach, our insights provide in-depth understanding of how online communities shape perceived support for cancer, but we cannot claim to provide generalisable results matched to all cancer survivors and their families. For instance, our sample was British and therefore we visited online communities within the context of patients and family members in the UK National Health Service (NHS). The UK NHS has particular pathways of care, which can have a resulting impact on the information which is believed to be pertinent to patients and family members.^56^ This may be influential since the use of cancer communities for information was crucial for the present sample. Additionally, the present study sample did not reflect diversity in regards to ethnicity as the sample were predominantly white participants. On the one hand, this is a typical failing of research which employs a volunteer recruitment strategy; research involving members of the minority communities in the co-design and recruitment may help to access these voices.^58^ On the other hand, there is no consensus of the demographic background of online cancer community users, and a limited understanding of whether support is experienced by British black and minority ethnic (BMAE) cancer populations when using online communities. Im and Chee speculated that virtual communities may be underused and inadequate for ethnic minority cancer survivors, who more commonly seek support from their family or religious groups.^59^ A review of British BMAE cancer populations has warned that a lack of use of support services did not necessarily reflect a lack of need for such services.^60^ Thus, even if use of online communities is limited, there is a need for future research to examine whether it could be a suitable resource if appropriately designed for the needs of these populations. This should be a key priority for future research, as we must ensure that we account for the support needs of typically underserved cancer populations.

## Conclusion

Defining what online cancer communities are is important, as existing policy regarding online peer support remains vague with limited guidance on which online cancer resources can offer peer and social support.^61^ Based on our findings, it appears important to steer people in need of peer support for cancer towards online groups and community-based resources with an emphasis on a sense of togetherness and dialogue. This study determined that online cancer communities can be distinguished by two types: open forum and secret groups. These differing groups have been characterised by features such as anonymity, reliability, exposure online, personalised interactions, a group hierarchy and the divide between the online and offline. Such features may interact with supportive online processes to provide a resource which can enhance support online, or burden individuals with their virtual exchanges. New and existing cancer support services wishing to capitalise on the digital revolution should closely consider the features which they wish to make available in order to foster a positive, supportive atmosphere for group members.

## References

[1] Ahmad AS Ormiston-Smith N Sasieni PD. Trends in the lifetime risk of developing cancer in Great Britain: Comparison of risk for those born from 1930 to 1960. Br J Cancer 2015; 112: 943–947. 25647015 10.1038/bjc.2014.606 PMC4453943

[2] Shaha M Cox CL Talman K et al Uncertainty in breast, prostate, and colorectal cancer: Implications for supportive care. J Nurs Scholarsh 2008; 40: 60–67. 18302593 10.1111/j.1547-5069.2007.00207.x

[3] Mitchell AJ Ferguson DW Gill J et al Depression and anxiety in long-term cancer survivors compared with spouses and healthy controls: A systematic review and meta-analysis. Lancet Oncol 2013; 14: 721–732. 23759376 10.1016/S1470-2045(13)70244-4

[4] Jaffee EM Van Dang C Agus DB et al Future cancer research priorities in the USA: A Lancet Oncology Commission. Lancet Oncol 2017; 18: e653–e706. 29208398 10.1016/S1470-2045(17)30698-8 PMC6178838

[5] National Institute for Clinical Excellence. Improving supportive and palliative care for adults with cancer: Executive summary. London: National Institute for Clinical Excellence, 2004.

[6] Jacobsen PB. Clinical practice guidelines for the psychosocial care of cancer survivors: Current status and future prospects. Cancer 2009; 115: 4419–4429. 19731353 10.1002/cncr.24589

[7] Recklitis CJ Syrjala KL. Provision of integrated psychosocial services for cancer survivors post-treatment. Lancet Oncol 2017; 18: e39–e50. 28049576 10.1016/S1470-2045(16)30659-3 PMC5865587

[8] Haslam C Jetten J Cruwys T et al The new psychology of health: Unlocking the social cure. London: Routledge, 2018.

[9] Gottlieb BH Wachala ED. Cancer support groups: A critical review of empirical studies. Psychooncology 2007; 16: 379–400. 16986205 10.1002/pon.1078

[10] Office for National Statistics. Internet users in the UK, https://www.ons.gov.uk/businessindustryandtrade/itandinternetindustry/bulletins/internetusers/2017 (2018, accessed 5 April 2019).

[11] PEW Research Centre. Internet/broadband fact sheet, http://www.pewinternet.org/fact-sheet/internet-broadband/ (2018, accessed 5 April 2019).

[12] Ziebland S. The importance of being expert: The quest for cancer information on the Internet. Soc Sci Med 2004; 59: 1783–1793. 15312914 10.1016/j.socscimed.2004.02.019

[13] Girault A Ferrua M Lalloué B et al Internet-based technologies to improve cancer care coordination: Current use and attitudes among cancer patients. Eur J Cancer 2015; 4: 551–557. 10.1016/j.ejca.2014.12.001 25661828

[14] An LC Wallner L Kirch MA. Online social engagement by cancer patients: A clinic-based patient survey. JMIR Cancer 2016; 2: e10. 28410186 10.2196/cancer.5785 PMC5369628

[15] Cao X Wang D. The role of online communities in reducing urban–rural health disparities in China. J Assoc Inf Sci Technol 2018; 69: 890–899.

[16] Suler J. The online disinhibition effect. Cyberpsychol Behav 2004; 7: 321–326. 15257832 10.1089/1094931041291295

[17] Barak A Grohol JM. Current and future trends in Internet-supported mental health interventions. J Technol Hum Serv 2011; 29: 155–196.

[18] Mo PK Coulson NS. Living with HIV/AIDS and use of online support groups. J Health Psychol 2010; 15: 339–350. 20348355 10.1177/1359105309348808

[19] White M Dorman SM. Receiving social support online: Implications for health education. Health Educ Res 2001; 16: 693–707. 11780708 10.1093/her/16.6.693

[20] van Eenbergen MC van de Poll-Franse LV Heine P et al The impact of participation in online cancer communities on patient reported outcomes: Systematic review. JMIR Cancer 2017; 3: e15. 28958985 10.2196/cancer.7312 PMC5639205

[21] Classen CC Chivers ML Urowitz S et al Psychosexual distress in women with gynecologic cancer: A feasibility study of an online support group. Psychooncology 2013; 22: 930–935. 22374732 10.1002/pon.3058

[22] Osei DK Lee JW Modest NN et al Effects of an online support group for prostate cancer survivors: A randomized trial. Urol Nurs 2013; 33: 123–134. 23930444

[23] Batenburg A Das E. Emotional coping differences among breast cancer patients from an online support group: A cross-sectional study. J Med Internet Res 2014; 16: e28. 24499687 10.2196/jmir.2831 PMC3936302

[24] Kim E Han JY Moon TJ et al The process and effect of supportive message expression and reception in online breast cancer support groups. Psychooncology 2012; 21: 531–540. 21416553 10.1002/pon.1942 PMC3168721

[25] van Eenbergen MC van de Poll-Franse LV Krahmer E et al Analysis of content shared in online cancer communities: Systematic review. JMIR Cancer 2018; 4: e6. 29615384 10.2196/cancer.7926 PMC5904449

[26] Hargreaves S Bath PA Duffin S et al Sharing and empathy in digital spaces: Qualitative study of online health forums for breast cancer and motor neuron disease (amyotrophic lateral sclerosis). J Med Internet Res 2018; 20: e222. 29903695 10.2196/jmir.9709 PMC6024105

[27] Erikainen S Pickersgill M Cunningham-Burley S et al Patienthood and participation in the digital era. Digital Health 2019; 5: 1–10. 10.1177/2055207619845546 PMC6482654 31041112

[28] Harkin LJ Beaver K Dey P et al Navigating cancer using online communities: A grounded theory of survivor and family experiences. J Cancer Surviv 2017; 11: 658–669. 28470506 10.1007/s11764-017-0616-1 PMC5671555

[29] Lepore SJ Buzaglo JS Lieberman MA et al Comparing standard versus prosocial Internet support groups for patients with breast cancer: A randomized controlled trial of the helper therapy principle. J Clin Oncol 2014; 32: 4081–4086. 25403218 10.1200/JCO.2014.57.0093 PMC4265118

[30] Bender JL Jimenez-Marroquin MC Jadad AR. Seeking support on Facebook: A content analysis of breast cancer groups. J Med Internet Res 2011; 13: e16. 21371990 10.2196/jmir.1560 PMC3221337

[31] Sugawara Y Narimatsu H Hozawa A et al Cancer patients on Twitter: A novel patient community on social media. BMC Res Notes 2012; 5: 699. 23270426 10.1186/1756-0500-5-699 PMC3599295

[32] Zhang S O’Carroll Bantum E Owen J et al Online cancer communities as informatics intervention for social support: Conceptualization, characterization, and impact. J Am Med Inform Assoc 2017; 24: 451–459. 27402140 10.1093/jamia/ocw093 PMC5565989

[33] Blank TO Schmidt SD Vangsness SA et al Differences among breast and prostate cancer online support groups. Comput Human Behav 2010; 26: 1400–1404.

[34] Durant KT McCray AT Safran C. Identifying gender-preferred communication styles within online cancer communities: A retrospective, longitudinal analysis. PloS One 2012; 7: e49169. 23155460 10.1371/journal.pone.0049169 PMC3498295

[35] Wang YC Kraut R Levine JM. To stay or leave? The relationship of emotional and informational support to commitment in online health support group. *Proceedings of the ACM 2012 Conference on Computer Supported Cooperative Work,* 11 February 2012, pp.833–842. Washington: Association for Computing Machinery.

[36] Braun V Clarke V. Using thematic analysis in psychology. Qual Res Psychol 2006; 3: 77–101.

[37] Braun V, Clarke V, Hayfield N, et al. Thematic analysis. In: Liamputtong P (ed.) *Handbook of Research Methods in Health Social Sciences*. 1st ed. Australia: Springer, 2019, pp. 843–860.

[38] Koskan A Klasko L Davis SN et al Use and taxonomy of social media in cancer-related research: A systematic review. Am J Public Health 2014; 104: e20–e37. 24832403 10.2105/AJPH.2014.301980 PMC4056246

[39] Taylor J Pagliari C. The social dynamics of lung cancer talk on Twitter, Facebook and Macmillan.org.uk. NPJ Digit Med 2019; 2: 51. 31304397 10.1038/s41746-019-0124-y PMC6557847

[40] Rodgers S Chen Q. Internet community group participation: Psychosocial benefits for women with breast cancer. J Comput Mediat Commun 2005; 10: e1047.

[41] Wakefield JR Bowe M Kellezi B et al When groups help and when groups harm: Origins, developments, and future directions of the ‘Social Cure’ perspective of group dynamics. Soc Personal Psychol Compass 2019; 13: e12440.

[42] Zhao S Grasmuck S Martin J. Identity construction on Facebook: Digital empowerment in anchored relationships. Comput Human Behav 2008; 24: 1816–1836.

[43] Boltong A Byrnes M McKiernan S et al Exploring the preferences, perceptions and satisfaction of people seeking cancer information and support: Implications for the Cancer Council helpline. Australian Journal of Cancer Nursing 2015; 16: 20–28.

[44] Scanlon K Carroll L. The role of Facebook and Twitter in signposting people affected by breast cancer to relevant information and support services: A case study of breast cancer care UK. Psychooncology 2013; 22: 16.

[45] Faller H Koch U Brähler E et al Satisfaction with information and unmet information needs in men and women with cancer. J Cancer Surviv 2016; 10: 62–70. 25956402 10.1007/s11764-015-0451-1

[46] Rutten LJ Arora NK Bakos AD et al Information needs and sources of information among cancer patients: A systematic review of research (1980–2003). Patient Educ Couns 2005; 57: 250–261. 15893206 10.1016/j.pec.2004.06.006

[47] Bernstam EV Walji MF Sagaram S et al Commonly cited website quality criteria are not effective at identifying inaccurate online information about breast cancer. Cancer 2008; 112: 1206–1213. 18266210 10.1002/cncr.23308

[48] Quinn EM Corrigan MA McHugh SM et al Who's talking about breast cancer? Analysis of daily breast cancer posts on the Internet. Breast 2013; 22: 24–27. 22683246 10.1016/j.breast.2012.05.001

[49] Macmillan Cancer Support. Facing the fight alone; isolation among cancer patients, http://www.macmillan.org.uk/Documents/AboutUs/MAC13970_Isolated_cancer_patients_media_reportFINAL.pdf (2013, accessed 5 April 2019).

[50] Bareham J. Facebook changes privacy settings after outing members of a closed medical support group, https://www.theverge.com/2018/7/12/17565754/facebook-brca-private-group-breast-cancer (2018, accessed 4 November 2019).

[51] Shane-Simpson C Manago A Gaggi N et al Why do college students prefer Facebook, Twitter, or Instagram? Site affordances, tensions between privacy and self-expression, and implications for social capital. Comput Human Behav 2018; 86: 276–288.

[52] Miller LMS Bell RA. Online health information seeking: The influence of age, information trustworthiness, and search challenges. J Aging Health 2012; 24: 525–541. 22187092 10.1177/0898264311428167

[53] Haslam SA Reicher S. Stressing the group: Social identity and the unfolding dynamics of responses to stress. J Appl Psychol 2006; 91: 1037–1052. 16953766 10.1037/0021-9010.91.5.1037

[54] Macmillan Cancer Support. Handbook for self-help and support groups, https://www.macmillan.org.uk/_images/Self%20help%20and%20support%20groups%20handbook%202017_tcm9-308337.pdf (2017, accessed 5 April 2019).

[55] Keeling D Khan A Newholm T. Internet forums and negotiation of healthcare knowledge cultures. J Serv Mark 2013; 27: 59–75.

[56] Maher J McConnell H. New pathways of care for cancer survivors: Adding the numbers. Br J Cancer 2011; 105: S5. 22048033 10.1038/bjc.2011.417 PMC3251951

[57] Costello L McDermott ML Wallace R. Netnography: Range of practices, misperceptions, and missed opportunities. Int J Qual Methods 2017; 16: 1.

[58] Yancey AK Ortega AN Kumanyika SK. Effective recruitment and retention of minority research participants. Annu Rev Public Health 2006; 27: 1–28. 16533107 10.1146/annurev.publhealth.27.021405.102113

[59] Im EO Chee W. The use of Internet cancer support groups by ethnic minorities. J Transcult Nurs 2008; 19: 74–82. 18217235 10.1177/1043659607309140

[60] Elkan R Avis M Cox K et al The reported views and experiences of cancer service users from minority ethnic groups: A critical review of the literature. Eur J Cancer Care (Engl) 2007; 16: 109–121. 17371419 10.1111/j.1365-2354.2006.00726.x

[61] World Health Organisation. Global strategy on digital health 2020–2024, https://extranet.who.int/dataform/upload/surveys/183439/files/Draft%20Global%20Strategy%20on%20Digital%20Health.pdf (2019, accessed 25 April 2019).

